# Semianalytical solution for the transient temperature in a scattering and absorbing slab consisting of three layers heated by a light source

**DOI:** 10.1038/s41598-021-87030-3

**Published:** 2021-04-19

**Authors:** Dominik Reitzle, Simeon Geiger, André Liemert, Alwin Kienle

**Affiliations:** Institut für Lasertechnologien in der Medizin und Meßtechnik an der Universität Ulm, Helmholtzstr. 12, 89081 Ulm, Germany

**Keywords:** Computational science, Applied physics, Optics and photonics

## Abstract

We derived a semianalytical solution for the time-dependent temperature distribution in a three-layered laterally infinite scattering and absorbing slab illuminated by an obliquely incident collimated beam of light. The light propagation was modeled by the low-order $P_1$ and $P_3$ approximations to the radiative transfer equation with closed form expressions for eigenvalues and eigenvectors, yielding a quickly computable solution, while the heat conduction was modeled by the Fourier equation. The solution was compared to a numerical solution using a Monte Carlo simulation for the light propagation and an FEM method for the heat conduction. The results showed that using the $P_3$ solution for the light propagation offers a large advantage in accuracy with only a moderate increase in calculation time compared to the $P_1$ solution. Also, while the $P_3$ solution is not a very good approximation for the spatially resolved absorbance itself, its application as a source term for the heat conduction equation does yield a very good approximation for the time-dependent temperature.

## Introduction

Non-contact thermal property measurement methods are widely used because they avoid contact resistances and require little to no sample preparation^1^. While there is a large variety of detection principles, most techniques use optical heating in the visual and near-infrared range. Specifically in photothermal radiometry (PTR), the detection is based on recording the thermal emission using an IR sensor. In the recent past, PTR has e.g. been used to retrieve microhardness profiles in case hardened steel^2^, detect partial curing in dental resins^3^ or study drug diffusion in human skin^4^. For semi-transparent, non-scattering media, Ravi et al. studied the reconstruction of absorption profiles using modulated PTR^5^, while Salazar et al. used the same method to simultaneously retrieve the absorption and thermal diffusivity profiles in semi-infinite media^6^ and multi-layered slabs^7^, utilizing analytical solutions of the heat conduction equation. Recently, Ren et al. extended this to temperature-dependent medium parameters for a 1D planar symmetric medium with coupled radiation and conduction using numerical solutions^8^, but also specifically excluded scattering. Apart from parameter retrieval with PTR, the required forward solutions can also be used to predict temperature distributions and, especially in biomedical applications, thermal damage after laser irradiation^9^. Here, as in many other media, light scattering is a large effect and therefore must be accounted for in a model for optical heating. The radiative transfer equation (RTE) is generally considered a very accurate model for light propagation on mesoscopic and macroscopic scales for scattering and absorbing media^10^. But due to its complexity, the RTE is often replaced by the diffusion equation (DE)^11,12^, which may be derived as an approximation to the RTE. Solutions to the DE are usually simple and can be computed quickly, but they are known to be inaccurate in many cases^13^. The $P_N$ approximations to the RTE^14^ on the other hand offer a much higher accuracy, but the computation time increases rapidly with the approximation order *N*. For low approximation orders *N* however, quickly computable closed form expressions can still be found^15^. While these low approximation order solutions can still exhibit large errors for the spatially resolved fluence or absorbance rate, their low spatial frequency components are already quite accurate. Using them as a source term for the heat conduction equation should therefore already produce highly accurate solutions for the medium temperature. The aim of this work therefore is to derive a semianalytical solution for the time-dependent temperature distribution in a three-layered scattering and absorbing medium heated by an incident beam of light, where the light propagation is modeled using low order $P_N$ approximation to the RTE. Specifically, we use the expansion orders $N = 1$, which leads to a diffusion-like approximation, and $N = 3$, for which closed form expressions are available for the homogeneous solution, resulting in short computation times.

In “Solution of the RTE” section, we present our light propagation model using the $P_N$ approximation to the RTE, including the used source and the optical boundary conditions for the three-layered medium. From this, we derive the source term for the heat conduction model, which is described in “Solution of the heat equation” section. Finally, in “Results and discussion” section, we compare the results of our solution to a purely numerical solution for a simple model of human skin.

## Solution of the RTE

### General solution

The steady-state radiative transport equation (RTE) governing the radiance $I(\mathbf {r},{\hat{\mathbf {s}}})$ at position $\mathbf {r}$ and direction ${\hat{\mathbf {s}}}$ for a point source at $\mathbf {r} = \mathbf {0}$ radiating in direction ${\hat{\mathbf {s}}}_0$ is given by

1 $$\begin{aligned} {\hat{\mathbf {s}}}\cdot \nabla I(\mathbf {r},{\hat{\mathbf {s}}}) + \mu _t I(\mathbf {r},{\hat{\mathbf {s}}}) - \mu _s \displaystyle \int _{{\mathbb {S}}^2} I(\mathbf {r},{\hat{\mathbf {s}}}\text{'}) f({\hat{\mathbf {s}}}\cdot {\hat{\mathbf {s}}}\text{'}) {\mathrm{d}}^2s\text{'} = \delta (\mathbf {r})\delta ({\hat{\mathbf{s}}}-{\hat{\mathbf {s}}}_0) \end{aligned}$$

with the phase function

2 $$\begin{aligned} f({\hat{\mathbf {s}}}\cdot {\hat{\mathbf {s}}}') = \sum _{l=0}^\infty \dfrac{2l+1}{4\pi }f_l P_l({\hat{\mathbf {s}}}\cdot {\hat{\mathbf {s}}}'), \end{aligned}$$

where $\mu _a$ is the absorption coefficient, $\mu _s$ is the scattering coefficient, $\mu _t = \mu _a + \mu _s$ is the attenuation coefficient, $f_l$ are the phase function moments and $P_l$ the Legendre polynomials. In order to improve the accuracy of low order $P_N$ approximations, we first approximate the phase function using the delta-M method^16,17^ of order *N* as

3 $$\begin{aligned} f({\hat{\mathbf {s}}}\cdot {\hat{\mathbf {s}}}') \approx \dfrac{f_{N+1}}{2\pi }\delta (1-{\hat{\mathbf {s}}}\cdot {\hat{\mathbf {s}}}') + \sum _{l=0}^N \dfrac{2l+1}{4\pi }(f_l-f_{N+1})P_l({\hat{\mathbf {s}}}\cdot {\hat{\mathbf {s}}}'). \end{aligned}$$

Next, we split a ballistic part off the radiance as $I = I_0 + I_d$. The ballistic part $I_0$ is then given by^18^

4 $$\begin{aligned} I_0(\mathbf {r},{\hat{\mathbf {s}}}) = \dfrac{e^{-{\tilde{\mu }}_t r}}{r^2} \delta ({\hat{\mathbf {r}}}-{\hat{\mathbf 
{s}}}_0)\delta ({\hat{\mathbf {s}}}-{\hat{\mathbf {s}}}_0), \end{aligned}$$

where the modified extinction coefficient becomes ${\tilde{\mu }}_t = \mu _a + (1-f_{N+1})\mu _s$. The diffuse radiance $I_d$ then satisfies the modified RTE

5 $$\begin{aligned} {\hat{\mathbf {s}}}\cdot \nabla I_d + {\tilde{\mu }}_t I_d - \mu _s \displaystyle \int _{{\mathbb {S}}^2} I_d \sum _{l=0}^N \dfrac{2l+1}{4\pi }(f_l-f_{N+1})P_l({\hat{\mathbf {s}}}\cdot {\hat{\mathbf {s}}}\text{'}) {\mathrm {d}}^2s\text{'} = S(\mathbf {r},{\hat{\mathbf{s}}},{\hat{\mathbf{s}}}_0), \end{aligned}$$

with the new source term

6 $$\begin{aligned} S(\mathbf {r},{\hat{\mathbf {s}}},{\hat{\mathbf {s}}}_0) = \mu _s \dfrac{e^{-{\tilde{\mu }}_t r}}{r^2} \delta ({\hat{\mathbf {r}}}-{\hat{\mathbf {s}}}_0) \sum _{l=0}^N \dfrac{2l+1}{4\pi }(f_l-f_{N+1})P_l({\hat{\mathbf {s}}}\cdot {\hat{\mathbf {s}}}_0). \end{aligned}$$

This new source corresponds to a line source in direction ${\hat{\mathbf {s}}}_0$ with exponentially decreasing fluence, radiating with the modified phase function rotated in beam direction. In the $P_N$ method, the diffuse radiance is expanded in spherical harmonics $Y_{lm}$ with expansion coefficients $\Psi _{lm}$ as

7 $$\begin{aligned} I_d(\mathbf {r},{\hat{\mathbf {s}}}) = \sum _{l=0}^N\sum _{m=-l}^l \Psi _{lm}(\mathbf {r}) Y_{lm}({\hat{\mathbf {s}}}). \end{aligned}$$

Using the transformed quantity

8 $$\begin{aligned} {\tilde{\Psi }}_{lm}(q,\phi _q,z) e^{-i m \phi _q} = \displaystyle \int _0^\infty \int _0^{2\pi } \Psi _{lm}(\rho ,\varphi ,z) e^{-i\rho q \cos (\varphi -\phi _q)} \rho \mathrm {d}\rho \mathrm {d}\varphi , \end{aligned}$$

the $P_N$ equations are then given by^15^

9 $$ \begin{aligned}&a_{l-1}^m \dfrac{\partial {\tilde{\Psi }}_{l-1,m}}{\partial z} + b_{l+1}^m \dfrac{\partial {\tilde{\Psi }}_{l+1,m}}{\partial z} + {\tilde{\sigma }}_l {\tilde{\Psi }}_{l,m} - \dfrac{iq}{2} \left( c_{l-1}^{m-1} {\tilde{\Psi }}_{l-1,m-1}- d_{l+1}^{m-1} {\tilde{\Psi }}_{l+1,m-1} \right) \\&\quad + \dfrac{iq}{2} \left( c_{l-1}^{-m-1} {\tilde{\Psi }}_{l-1,m+1} - d_{l+1}^{-m-1} {\tilde{\Psi }}_{l+1,m+1} \right) = E_{lm}(q,\phi _q,z), \end{aligned}$$

for $0\le m \le N$ and $m \le l \le N$ with the condition

10 $$\begin{aligned} {\tilde{\Psi }}_{l,-m} = (-1)^m {\tilde{\Psi }}_{l,m}, \end{aligned}$$

where the right hand side $E_{lm}$ must be determined from the source term (see Eq. (15)). The coefficients are given by^15^

11 $$\begin{aligned} {\tilde{\sigma }}_l = {\tilde{\mu }}_t - (f_l - f_{N+1})\mu _s = \mu _t - f_l \mu _s = \sigma _l \end{aligned}$$

and

12 $$\begin{aligned} a_l^m&= \sqrt{\dfrac{(l-m+1)(l+m+1)}{(2l+1)(2l+3)}} , ~~ b_l^m = \sqrt{\dfrac{(l-m)(l+m)}{(2l-1)(2l+1)}} ,\\ c_l^m&= \sqrt{\dfrac{(l+m+1)(l+m+2)}{(2l+1)(2l+3)}} , ~~ d_l^m = \sqrt{\dfrac{(l-m)(l-m-1)}{(2l-1)(2l+1)}}. \end{aligned}$$

Note that the rotation $e^{-i m \phi _q}$ in Eq. (8) makes the left hand side of the system (9) independent of $\phi _q$, allowing a much more efficient numerical evaluation. The $\sigma _l$ in Eq. (11) are the coefficients that follow directly from applying the $P_N$ method to Eq. (1) without the delta-M method and the ballistic part separation^14^. It is therefore evident that the homogeneous solutions of Eqs. (1) and (5) are identical. These solutions can for example be found using the method of rotated reference frames, which was studied intensively over the last few years. The solution for the diffuse radiance then has the form^14^

13 $$ \begin{aligned} I_d(q,\phi _q,z,\theta ,\varphi )&= \displaystyle \sum _{l=0}^N\sum _{m=-l}^l\left[ \sum _i C_i(q,\phi _q)e^{-\sqrt{q^2+\frac{1}{\xi _i^2}}z} \Lambda _{lm}^i(q) \right. \\&\left. \quad + \sum _i C_i'(q,\phi _q)e^{\sqrt{q^2+\frac{1}{\xi _i^2}}z} (-1)^l \Lambda _{lm}^i(q) + {\tilde{\Psi }}_{lm}^{(p)}(q,\phi _q,z) \right] Y_{lm}(\theta ,\varphi -\phi _q). \end{aligned} $$

Here, $C_i$ and $C_i'$ are unknown coefficients determined by the boundary conditions, $\sqrt{q^2+\frac{1}{\xi _i^2}} =: \zeta _i(q)$ are the positive eigenvalues and $\Lambda _{lm}^i(q)$ the corresponding eigenvector components. We refer the reader to previous publications^14,15,19–21^ for details on this method.

For the particular solution ${\tilde{\Psi }}_{lm}^{(p)}(q,\phi _q,z)$ with the source term (6), we first calculate its spherical harmonics decomposition

14 $$\begin{aligned} \epsilon _{lm}(\mathbf {r}) = \displaystyle \int _{{\mathbb {S}}^2} S(\mathbf {r},{\hat{\mathbf{s}}},{\hat{\mathbf{s}}}_0) Y_{lm}^*({\hat{\mathbf{s}}}){\mathrm {d}}^2s = \mu _s \dfrac{e^{-{\tilde{\mu }}_t r}}{r^2} \delta ({\hat{\mathbf{r}}}-{\hat{\mathbf{s}}}_0)(f_l-f_{N+1}) Y_{lm}^*({\hat{\mathbf{s}}}_0). \end{aligned}$$

Applying a 2D Fourier transform, setting ${\hat{\mathbf {s}}}_0 = (\theta _0,0)$ in spherical coordinates and making use of condition (10), we obtain the desired moments

15 $$\begin{aligned} E_{lm}(q,\phi _q,z) = \dfrac{\mu _s}{|\mu _0|}\Theta (\frac{z}{\mu _0})e^{-\frac{{\tilde{\mu }}_t}{\mu _0}z} e^{-iqz\tan (\theta _0)\cos (\phi _q)}(f_l-f_{N+1}) \mathfrak {R}\left\{ Y_{lm}(\theta _0,\phi _q)\right\} , \end{aligned}$$

with $\mu _0 = \cos (\theta _0)$. Introducing the abbreviation^22^

16 $$\begin{aligned} \mu _c = \frac{{\tilde{\mu }}_t}{\mu _0} + iq \tan (\theta _0)\cos (\phi _q), \end{aligned}$$

the similarity ansatz ${\tilde{\Psi }}_{lm}^{(p)} = \chi _{lm} e^{-\mu _c z}$ can be used to solve the system (9) with the source moments (15), resulting in the system of equations for $z \ge 0$ and $1 \ge \mu _0 > 0$

17 $$\begin{aligned} -\mu _c \left( a_{l-1}^m \chi _{l-1,m} + b_{l+1}^m \chi _{l+1,m} \right) + \sigma _l \chi _{l,m} - \dfrac{iq}{2} \left( c_{l-1}^{m-1} \chi _{l-1,m-1} - d_{l+1}^{m-1} \chi _{l+1,m-1} \right) \\ + \dfrac{iq}{2} \left( c_{l-1}^{-m-1} \chi _{l-1,m+1} - d_{l+1}^{-m-1} \chi _{l+1,m+1} \right) = \dfrac{\mu _s}{\mu _0} (f_l-f_{N+1}) \mathfrak {R}\left\{ Y_{lm}(\theta _0,\phi _q)\right\} . \end{aligned}$$

In many cases, only the fluence rate $\Phi (q,\phi _q,z) = \int _{{\mathbb {S}}^2} I(q,\phi _q,z,{\hat{\mathbf {s}}}) \mathrm {d}^2s$ is required instead of the radiance from Eqs. (4) and (13). Using the same set of polar coordinates for the ballistic part, the fluence rate is found to be

18 $$\begin{aligned} \Phi _0(q,\phi _q,z)= & {} \dfrac{1}{|\mu _0|}\Theta \left( \frac{z}{\mu _0}\right) e^{-\mu _c z} , \end{aligned}$$

19 $$\begin{aligned} \Phi _d(q,\phi _q,z)= & {} \sqrt{4\pi }\displaystyle \sum _i \left[ C_i(q,\phi _q)e^{-\zeta _i(q) z} + C_i'(q,\phi _q)e^{\zeta _i(q) z} \right] \Lambda _{00}^i(q) +\sqrt{4\pi } \chi _{00}(q,\phi _q) e^{-\mu _c z}. \end{aligned}$$

### RTE boundary conditions for a three-layered slab

**Figure 1:**
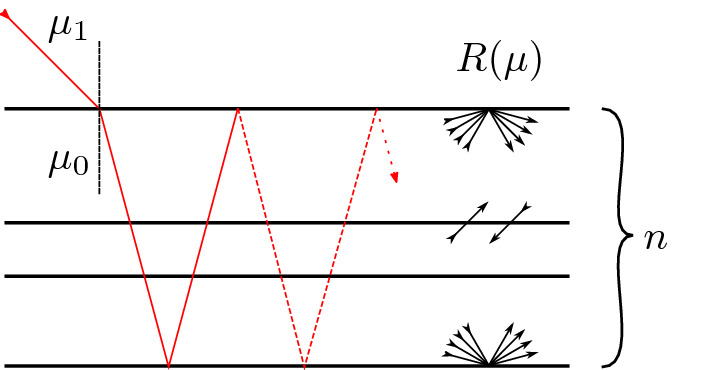
Schematic of the problem geometry. A stack of three laterally infinite layers is illuminated by a collimated beam with angle cosines of $\mu _1$ and $\mu _0$ outside and inside of the stack, respectively. On the top and bottom surface, Fresnel reflections must be accounted for, while the radiance is continuous at the layer interfaces.

Our goal here is to construct a solution for a stack of three laterally infinite layers, where for simplicity all layers share the same refractive index. The refractive index outside the stack may, however, be different from the one inside so that Fresnel reflection at the top and bottom surfaces must be accounted for in the boundary conditions. Figure 1 illustrates this problem geometry schematically.

To that end, we first rescale the coefficients $C_i$ and $C_i'$ from equation (13) for each layer $n \in \{1,2,3\}$ with thickness $L^{(n)}$ and take the extinction and shift *D*(*n*) of the incident light by the layers above into account, resulting in

20 $$\begin{aligned} I_d^{(n)}(q,\phi _q,z,\theta ,\varphi )&= \displaystyle \sum _{l=0}^N\sum _{m=-l}^l\left[ \sum _i C_i^{(n)}(q,\phi _q)e^{-\zeta ^{(n)}_i(q)(z-\lambda ^{(n)})} \Lambda _{lm}^{i,(n)}(q) \right. \\&\left. \quad + \sum _i C'^{(n)}_i(q,\phi _q)e^{\zeta ^{(n)}_i(q)(z-\lambda ^{(n)}-L^{(n)})} (-1)^l \Lambda _{lm}^{i,(n)}(q) \right. \\&\left. \quad + \chi ^{(n)}_{lm}(q,\phi _q,z) e^{-\mu _c^{(n)}(z-\lambda ^{(n)})} D(n) \right] Y_{lm}(\theta ,\varphi -\phi _q), \end{aligned}$$

where we defined

21 $$\begin{aligned} \lambda ^{(n)} = \displaystyle \sum _{k=1}^{n-1} L^{(k)}, \end{aligned}$$

and

22 $$\begin{aligned} D(n) = \displaystyle \prod _{k=1}^{n-1} e^{-\mu _c^{(k)} L^{(k)}}. \end{aligned}$$

Using $\mu = \cos (\theta )$ and $\mu >0$, the boundary conditions are given by^13^

23 $$\begin{aligned}&I^{(1)}(z=0,\mu ,\varphi ) = R(\mu ) I^{(1)}(z=0,-\mu ,\varphi ), \end{aligned}$$

24 $$\begin{aligned}&I^{(3)}(z=\lambda ^{(4)},-\mu ,\varphi ) = R(\mu ) I^{(3)}(z=\lambda ^{(4)},\mu ,\varphi ), \end{aligned}$$

25 $$\begin{aligned}&I^{(n)}(z=\lambda ^{(n+1)},\mu ,\varphi ) = I^{(n+1)}(z=\lambda ^{(n+1)},\mu ,\varphi ) ,~~n=\{1,2\}, \end{aligned}$$

26 $$\begin{aligned}&I^{(n)}(z=\lambda ^{(n+1)},-\mu ,\varphi ) = I^{(n+1)}(z=\lambda ^{(n+1)},-\mu ,\varphi ) ,~~n=\{1,2\}, \end{aligned}$$

where $R(\mu )$ is the Fresnel reflection coefficient for the top and bottom surfaces^23^. Multiplying all conditions with $Y^*_{l'm'}(\mu ,\varphi )$ and integrating over the half-space $\mu >0$ then yields the generalized Marshak boundary conditions^14,22,24^. Suppressing the arguments from Eq. (20), we get from Eq. (23) for the top surface

27 $$\begin{aligned} \displaystyle \sum _l R_{l' l}^{m'} \left( \sum _i C_i^{(1)} \Lambda _{lm'}^{i,(1)} + \sum _i C'^{(1)}_i e^{-\zeta ^{(1)}_i L^{(1)}} (-1)^l \Lambda _{lm'}^{i,(1)} \right) = -\sum _l R_{l' l}^{m'} \chi ^{(3)}_{lm'}, \end{aligned}$$

from Eq. (24) for the bottom surface

28 $$\begin{aligned} \displaystyle \sum _l R_{l' l}^{m'} \left( \sum _i C_i^{(3)} e^{-\zeta ^{(3)}_i L^{(3)}} (-1)^l \Lambda _{lm'}^{i,(3)} + \sum _i C'^{(3)}_i \Lambda _{lm'}^{i,(3)} \right) = -\sum _l R_{l' l}^{m'} (-1)^l D(4) \chi ^{(3)}_{lm'}, \end{aligned}$$

and from Eqs. (25) and (26) with $n=\{1,2\}$ for the interfaces between the layers

29 $$\begin{aligned} &\displaystyle \sum _l T_{l' l}^{m'} \left( \sum _i C_i^{(n)} e^{-\zeta ^{(n)}_i L^{(n)}} \Lambda _{lm'}^{i,(n)} + \sum _i C'^{(n)}_i (-1)^l \Lambda _{lm'}^{i,(n)} - \sum _i C^{(n+1)}_i \Lambda _{lm'}^{i,(n+1)} \right. \\&\quad \left. - \sum _i C'^{(n+1)}_i e^{-\zeta ^{(n+1)}_i L^{(n+1)}} (-1)^l \Lambda _{lm'}^{i,(n+1)} \right) = \sum _l T_{l' l}^{m'} D(n+1) \left( \chi _{l m'}^{(n+1)} - \chi _{l m'}^{(n)} \right) , \end{aligned}$$

and

30 $$\begin{aligned}&\displaystyle \sum _l T_{l' l}^{m'} (-1)^l \left( \sum _i C_i^{(n)} e^{-\zeta ^{(n)}_i L^{(n)}} \Lambda _{lm'}^{i,(n)} + \sum _i C'^{(n)}_i (-1)^l \Lambda _{lm'}^{i,(n)} - \sum _i C^{(n+1)}_i \Lambda _{lm'}^{i,(n+1)} \right. \\&\quad \left. - \sum _i C'^{(n+1)}_i e^{-\zeta ^{(n+1)}_i L^{(n+1)}} (-1)^l \Lambda _{lm'}^{i,(n+1)} \right) = \sum _l T_{l' l}^{m'} (-1)^l D(n+1) \left( \chi _{l m'}^{(n+1)} - \chi _{l m'}^{(n)} \right) . \end{aligned}$$

Here, the coefficients $R_{l l'}^{m'}$ for the Fresnel reflection at the top and bottom surfaces are given by^14,21^

31 $$\begin{aligned} R_{l l'}^{m'} = \sqrt{\dfrac{(2l+1)(2l'+1)(l-m')!(l'-m')!}{(l+m')!(l'+m')!}} \times \displaystyle \int _0^1\left[ 1-(-1)^{l+m'}R(\mu )\right] P_l^{m'}(\mu )P_{l'}^{m'}(\mu ) \mathrm {d}\mu , \end{aligned}$$

while the transmission coefficients $T_{l l'}^{m'}$ for the boundaries between layers are

32 $$\begin{aligned} T_{l l'}^{m'} = \sqrt{\dfrac{(2l+1)(2l'+1)(l-m')!(l'-m')!}{(l+m')!(l'+m')!}} \times \displaystyle \int _0^1 P_l^{m'}(\mu )P_{l'}^{m'}(\mu ) \mathrm {d}\mu . \end{aligned}$$

Following the standard prescription for the generalized Marshak boundary conditions^14^ by picking for each $l' = 1,\ldots ,N$ the Eqs. (27)–(30) with $m' = l'-1,l'-3,\ldots$ results in a system of equations that determines all unknown coefficients $C_i^{(n)}$ and $C'^{(n)}_i$, completing the solution for the $P_N$ approximation to the RTE. In all of the above, a single source with unit strength just below the upper boundary was assumed. To model an incident collimated beam from outside the medium, we have to take the Fresnel reflection outside the medium as well as internal reflections of the unscattered light into account. If $R_d(\mu _1)$ is the reflection coefficient outside the medium and $R_0 = R(\mu _0)$ the reflection coefficient inside the medium, where $\mu _1$ and $\mu _0$ are connected by Snell’s law, the sources due to multiple internal reflections can be reduced to two sources with strengths $S_o$ and $S_u$ for the top and bottom surface, respectively, with

33 $$\begin{aligned} S_o= & {} (1-R_d)\displaystyle \sum _{n=0}^\infty \left( R_0^2 D(4)^2 \right) ^n = \dfrac{1-R_d}{1-R_0^2 D(4)^2}, \end{aligned}$$

34 $$\begin{aligned} S_u= & {} R_0 D(4) S_o. \end{aligned}$$

In many cases, $R_0 D(4)$ is very small and the lower source may be neglected. If however the medium is optically thin enough for the lower source to matter, the coefficients $C_i^{(n)}$ and $C'^{(n)}_i$ must be recalculated with reversed layer order for a complete solution.

## Solution of the heat equation

We assume that the heat transport inside the medium is governed by the differential equation

35 $$\begin{aligned} \rho ^{(n)} c_p^{(n)} \dfrac{\partial T(\mathbf {r},t)}{\partial t}- k^{(n)} \Delta T(\mathbf {r},t) = \delta _{nm}Q_s(x,y)\delta (z-z_0)Q_t(t)\Theta (t) \end{aligned}$$

for the temperature $T(\mathbf {r},t)$, where $\rho$ is the density, $c_p$ the heat capacity and *k* the isotropic thermal conductivity. *n* is the layer number and *m* the layer containing a source sheet at depth $z_0$ with spatial profile $Q_s(x,y)$ and time dependence $Q_t(t)\Theta (t)$. For the heat equation (35) to be valid, the spectral components of $Q_t(t)$ must be restricted to frequencies well below 1 GHz^25^. This allows using the steady-state RTE absorbance for the spatial source profile. Also, the layers should be at least several micrometers thick. Applying the same 2*D*-Fourier transform we used for the RTE and a Laplace transform with respect to time to (35) using $T=0$ everywhere as initial condition, we get with the Laplace variable *s*

36 $$\begin{aligned} \left( \dfrac{\partial ^2}{\partial z^2} - (\alpha ^{(n)})^2 \right) {\tilde{T}}^{(n)}(s,q,\phi _q,z) = -\delta _{nm}\dfrac{1}{k^{(n)}}{\tilde{Q}}_s(q,\phi _q){\tilde{Q}}_t(s)\delta (z-z_0) , \end{aligned}$$

with

37 $$\begin{aligned} \alpha ^{(n)} = \sqrt{\dfrac{\rho ^{(n)} c_p^{(n)}}{k^{(n)}} s + q^2} , \end{aligned}$$

where the tilde marks transformed quantities. We note that in our previous work^26^, we derived a more general solution of the heat conduction equation for *N* layers with anisotropic thermal conductivities. The solution we require here, therefore, arises as a special case and is given by

38 $$\begin{aligned} {\tilde{T}}^{(n)}_m(s,q,\phi _q,z,z_0)= & {} A^{(n)}_m e^{\alpha ^{(n)} (z-\lambda ^{(n)}-L^{(n)})} + B^{(n)}_m e^{-\alpha ^{(n)} (z-\lambda ^{(n)})} + \delta _{nm} {\tilde{P}}^{(m)}(s,q,\phi _q,z,z_0), \end{aligned}$$

39 $$\begin{aligned} {\tilde{P}}^{(m)}(s,q,\phi _q,z,z_0)= & {} \dfrac{{\tilde{Q}}_s(q,\phi _q){\tilde{Q}}_t(s)}{2k^{(m)} \alpha ^{(m)}}e^{-\alpha ^{(m)}|z-z_0|}. \end{aligned}$$

The coefficients $A^{(n)}_m$ and $B^{(n)}_m$ are determined by the boundary conditions for the heat equation, where we used Robin-type convective boundary conditions for the upper and lower boundaries and the perfect thermal contact between layers, resulting in a system of equations for each *m*. For the special case of three layers, we arrive at

40 $$\begin{aligned} {\mathbf {M}}\cdot \begin{pmatrix} A^{(1)}_m\\ B^{(1)}_m\\ A^{(2)}_m\\ B^{(2)}_m\\ A^{(3)}_m\\ B^{(3)}_m \end{pmatrix} = \begin{pmatrix} -\delta _{m1} \frac{1}{k^{(1)}} f_2^{(0)} (1-\frac{h_1}{k^{(1)}\alpha ^{(1)}})\\ -\delta _{m1} \frac{1}{k^{(1)}\alpha ^{(1)}} f_1^{(1)} + \delta _{m2} \frac{1}{k^{(2)}\alpha ^{(2)}} f_2^{(1)} \\ \delta _{m1} f_1^{(1)} + \delta _{m2} f_2^{(1)} \\ -\delta _{m2} \frac{1}{k^{(2)}\alpha ^{(2)}} f_1^{(2)} + \delta _{m3} \frac{1}{k^{(3)}\alpha ^{(3)}} f_2^{(2)} \\ \delta _{m2} f_1^{(2)} + \delta _{m3} f_2^{(2)} \\ \delta _{m3} \frac{1}{k^{(3)}} f_1^{(3)} (1-\frac{h_2}{k^{(3)}\alpha ^{(3)}}) \end{pmatrix}, \end{aligned}$$

with

41 $$\begin{aligned} f_1^{(m)}&= \dfrac{{\tilde{Q}}_s(q,\phi _q){\tilde{Q}}_t(s)}{2} e^{-\alpha ^{(m)}(\lambda ^{(m+1)}-z_0)}, \end{aligned}$$

42 $$\begin{aligned} f_2^{(m)}&= \dfrac{{\tilde{Q}}_s(q,\phi _q){\tilde{Q}}_t(s)}{2} e^{-\alpha ^{(m)}(z_0-\lambda ^{(m+1)})}. \end{aligned}$$

The matrix ${\mathbf {M}}$ is given in^26^ and in Appendix A. In^26^, we gave closed form expressions for the 6 coefficients $A^{(n)}_1$ and $B^{(n)}_1$ for a three-layered system and sources in the first layer. Since in our case there are sources in all layers, we also need the expressions for the remaining 12 coefficients. For sources in the third layer, they follow immediately from the coefficients for sources in the first layer with reversed layer properties. The coefficients $A^{(n)}_2$ and $B^{(n)}_2$ for sources in the second layer, however, must be calculated separately and are given in Appendix A. The spatial source distribution ${\tilde{Q}}_s(q,\phi _q)$ at depth $z_0$ is now given by the corresponding RTE absorbance

43 $$\begin{aligned} {\tilde{Q}}_s(q,\phi _q, z_0) = \mu _a^{(m)} \Phi ^{(m)}(q,\phi _q,z_0) , \end{aligned}$$

with the fluence

44 $$\begin{aligned}  \Phi ^{(m)}(q,\phi _q,z_0)&= \sqrt{4\pi }\displaystyle \sum _i \Lambda _{00}^{i,(m)}(q) \left[ C^{(m)}_i(q,\phi _q)e^{-\zeta ^{(m)}_i(q) (z_0-\lambda ^{(m)})} + C'^{(m)}_i(q,\phi _q)e^{\zeta ^{(m)}_i(q) (z_0-\lambda ^{(m)}-L^{(m)})} \right] \\&\quad +\left( \sqrt{4\pi } \chi ^{(m)}_{00}(q,\phi _q) + \frac{1}{|\mu _0|}\right) D(m) e^{-\mu _c^{(m)} (z-\lambda ^{(m)})}. \end{aligned}$$

With the contributions from all depths known, we can then calculate the complete solution as

45 $$\begin{aligned} {\tilde{T}}^{(n)}(s,q,\phi _q,z) = \displaystyle \sum _m\int _{\lambda ^{(m)}}^{\lambda ^{(m+1)}} {\tilde{T}}_m^{(n)}(s,q,\phi _q,z,z_0) \mathrm {d}z_0. \end{aligned}$$

Looking at Eqs. (38) and (39), the integral in Eq. (45) for a fixed *m* only acts on the coefficients $f_1^{(m)}$ and $f_2^{(m-1)}$ and on the particular solution. We can, therefore, solve the integrals and obtain a closed form solution by replacing $f_1^{(m)}$ and $f_2^{(m-1)}$ in the solution of (40) with ${\bar{f}}_1^{(m)}$ and ${\bar{f}}_2^{(m-1)}$ as

46 $$\begin{aligned}  {\bar{f}}_1^{(m)}&= \dfrac{\mu _a^{(m)}{\tilde{Q}}_t(s)}{2} \Bigg \{ \sqrt{4\pi } \displaystyle \sum _i \Lambda _{00}^{i,(m)} \left[ C^{(m)}_i \dfrac{e^{-\alpha ^{(m)} L^{(m)}}-e^{-\zeta ^{(m)}_i L^{(m)}}}{\zeta ^{(m)}_i-\alpha ^{(m)}} + C'^{(m)}_i \dfrac{1-e^{-(\alpha ^{(m)}+\zeta ^{(m)}_i)L^{(m)}}}{\zeta ^{(m)}_i+\alpha ^{(m)}} \right] \\&\quad + \left( \sqrt{4\pi }\chi ^{(m)}_{00} + \dfrac{1}{\mu _0}\right) D(m) \dfrac{e^{-\mu _c^{(m)}L^{(m)}}-e^{-\alpha ^{(m)}L^{(m)}}}{\alpha ^{(m)}-\mu _c^{(m)}}\Bigg \} , \end{aligned}$$

and

47 $$\begin{aligned}  {\bar{f}}_2^{(m-1)}&= \dfrac{\mu _a^{(m)}{\tilde{Q}}_t(s)}{2} \left\{ \sqrt{4\pi } \displaystyle \sum _i \Lambda _{00}^{i,(m)} \left[ C^{(m)}_i \dfrac{1-e^{-(\alpha ^{(m)}+\zeta ^{(m)}_i)L^{(m)}}}{\zeta ^{(m)}_i+\alpha ^{(m)}} + C'^{(m)}_i \dfrac{e^{-\alpha ^{(m)} L^{(m)}}-e^{-\zeta ^{(m)}_i L^{(m)}}}{\zeta ^{(m)}_i-\alpha ^{(m)}} \right] \right. \\&\quad \left. + \left( \sqrt{4\pi }\chi ^{(m)}_{00} + \dfrac{1}{\mu _0}\right) D(m) \dfrac{1-e^{-(\alpha ^{(m)}+\mu _c^{(m)})L^{(m)}}}{\alpha ^{(m)}+\mu _c^{(m)}}\right\} , \end{aligned}$$

and replacing the particular solution (39) with ${\bar{P}}^{(m)}(s,q,\phi _q,z)$ for $\lambda ^{(m)} \le z \le \lambda ^{(m+1)}$ as

48 $$\begin{aligned} {\bar{P}}^{(m)}(s,q,\phi _q,z)&= \dfrac{\mu _a^{(m)}{\tilde{Q}}_t(s)}{2k^{(m)}\alpha ^{(m)}} \left\{ \sqrt{4\pi } \displaystyle \sum _i \dfrac{\Lambda _{00}^{i,(m)}}{(\alpha ^{(m)})^2-(\zeta ^{(m)}_i)^2} \right. \\&\quad \left. \times \left[ C^{(m)}_i \left( 2\alpha ^{(m)} e^{-\zeta ^{(m)}_i(z-\lambda ^{(m)})} + (\zeta ^{(m)}_i-\alpha ^{(m)}) e^{-\zeta ^{(m)}_i L^{(m)}}e^{-\alpha ^{(m)}(\lambda ^{(m+1)}-z)} \right. \right. \right. \\&\quad \left. \left. \left. - (\zeta ^{(m)}_i + \alpha ^{(m)}) e^{-\alpha ^{(m)}(z-\lambda ^{(m)})} \right) + C'^{(m)}_i \left( 2\alpha ^{(m)} e^{-\zeta ^{(m)}_i(\lambda ^{(m+1)}-z)} \right. \right. \right. \\&\left. \left. \left. \quad + (\zeta ^{(m)}_i-\alpha ^{(m)}) e^{-\zeta ^{(m)}_i L^{(m)}}e^{-\alpha ^{(m)}(z-\lambda ^{(m)})} - (\zeta ^{(m)}_i + \alpha ^{(m)}) e^{-\alpha ^{(m)}(\lambda ^{(m+1)}-z)} \right) \right] \right. \\&\quad \left. + \left( \sqrt{4\pi }\chi ^{(m)}_{00} + \dfrac{1}{\mu _0} \right) \dfrac{D(m)}{(\alpha ^{(m)})^2-(\mu _c^{(m)})^2} \left[ 2\alpha ^{(m)} e^{-\mu _c^{(m)}(z-\lambda ^{(m)})} \right. \right. \\&\quad \left. \left. + (\mu _c^{(m)}-\alpha ^{(m)}) e^{-\mu _c^{(m)} L^{(m)}}e^{-\alpha ^{(m)}(\lambda ^{(m+1)}-z)} - (\mu _c^{(m)} + \alpha ^{(m)}) e^{-\alpha ^{(m)}(z-\lambda ^{(m)})} \right] \right\} . \end{aligned}$$

## Results and discussion

In this section, the obtained solution is tested and validated against a purely numerical solution. As an exemplary system, we choose a three-layered generic model of human tissue in the near-infrared range^27,28^. The three layers represent skin, subcutaneous fat and muscle tissue, respectively. The thermal parameters of the model are listed in Table 1 and the optical parameters in Table 2. For all layers, we assume a refractive index of $n_i=1.4$ with $n_o=1.0$ outside the medium and the Henyey–Greenstein phase function with $g=0.8$ using the moments $f_l = g^l$. For the upper boundary, we use a heat transfer coefficient of $h_1 = 100\,\hbox {W\,m}^{-2}\,\hbox {K}^{-1}$ with an ambient temperature of $T_a=0$ equal to the initial medium temperature and assume an adiabatic lower boundary with $h_2 = 0$.

**Table 1:** Thermal parameters of the considered medium.

	$\rho (\hbox {kg\,m}^{-3})$	$c_p (\hbox {J\,kg}^{-1}\,\hbox {K}^{-1})$	$k(\hbox {W\,m}^{-1}\,\hbox {K}^{-1})$	*l* (mm)
1	1100	3000	0.25	1
2	900	3000	0.2	2
3	1100	3500	0.5	10

**Table 2:** Optical parameters of the considered medium. For the phase function, the Henyey–Greenstein function is used. The refractive index $n_i$ must be identical for all layers, while the refractive index outside the medium is set to 1.0.

	$\mu _s' (\hbox {mm}^{-1})$	$\mu _a (\hbox {mm}^{-1})$	*g*	$n_i$
1	2.0	0.02	0.8	1.4
2	1.0	0.003	0.8	1.4
3	0.5	0.04	0.8	1.4

As an input beam, we use a Gaussian beam with beam radius $r_w = 1\,\hbox {mm}$ hitting the top surface with an angle outside the medium of $\mu _1 = \cos (\theta _1)$, centered at the origin. We use $\theta _1 = 0^\circ$ and $\theta _1 = 60^\circ$ as incidence angles. Taking the distortion of the beam profile due to the oblique incidence into account, the input beam profile is then given by^22^

49 $$\begin{aligned} f(x,y) = \dfrac{2\mu _1}{\pi r_w^2} \exp \left\{ -2\dfrac{\mu _1^2x^2+y^2}{r_w^2}\right\} . \end{aligned}$$

Applying a 2D Fourier transform to (49) again yields

50 $$\begin{aligned} {\tilde{F}}(q,\phi _q) = \exp \left\{ -\frac{1}{8} r_w^2 q^2\left( \sin ^2(\phi _q) + \frac{1}{\mu _1^2}\cos ^2(\phi _q) \right) \right\} . \end{aligned}$$

The lower source strength from Eq. (34) depends on the approximation order and on the angle of incidence, but even for normal incidence with the $P_1$ approximation, it is nine orders of magnitude weaker than the upper source. Therefore, we can safely neglect the lower source in all calculations. As approximation orders, we consider only the $P_1$ and $P_3$ approximations, because for these, there are closed form expressions available for the eigenvalues and eigenvectors. For the $P_3$, we use the expressions given by Liemert et al.^15^, while the $P_1$ expressions are given in Appendix B. Although theoretically possible up to $P_7$, no higher order closed form expressions are available to the authors’ knowledge. Computing the eigenvalues and eigenvectors numerically is possible for higher orders, but computationally quite expensive. Also, the solution would become more and more unstable for high values of *q*, which would render the numerical inverse Fourier transform extremely tricky. To obtain a numerical solution to compare to, we use a two step process. First, the light propagation is calculated using a custom GPU-accelerated Monte Carlo solver, recording the spatially resolved fluence rate inside the medium. Then, we use the COMSOL Multiphysics software^29^ to interpolate the resulting absorbance from the Monte Carlo simulation and calculate the time-dependent temperature rise.

**Figure 2:**
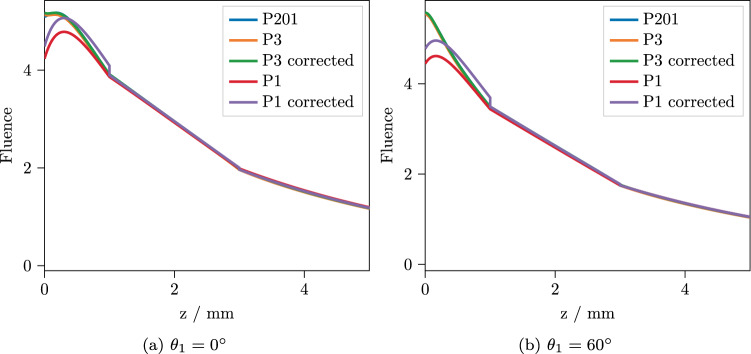
Depth-resolved fluence rate for the three-layered system, integrated over *x* and *y*. The $P_{201}$ solution can be regarded as practically exact. For the corrected solutions, the total absorbance per layer was matched to the $P_{201}$ solution.

**Table 3:** Total absorbance error for each layer of the $P_1$ and $P_3$ approximations relative to a $P_{201}$ approximation that may be regarded as an exact solution.

		Layer 1	Layer 2	Layer 3
*P*1	$0^{\circ }$	$5.925\%$	$0.7962\%$	−1.035%
	$60^{\circ }$	$7.428\%$	$1.534\%$	0.0275%
*P*3	$0^{\circ }$	$0.7325\%$	$0.7978\%$	0.8433%
	$60^{\circ }$	$0.7000\%$	$0.8089\%$	0.8538%

Since we expect the $P_N$ approximation to be the main source of error in the final results, we first look at the predicted fluence rate. Figure 2 contains the depth-resolved total fluence rate calculated in $P_1$ and $P_3$ approximation, integrated over *x* and *y*. Here, we do not yet have to resort to Monte Carlo methods to obtain a correct reference, since for planar symmetry or $q=0$, the $P_N$ equations can be solved efficiently for high orders *N*. Integrating the depth resolved total fluence rate over a layer and multiplying by the respective $\mu _a$ yields the total absorbance inside that layer. As the dependence on *z* of the fluence rate is very simple (compare Eqs. (18) and (19)), this integration can be done analytically and these total absorbances per layer can, therefore, be calculated with little computational effort. Table 3 contains the errors of the total absorbances per layer for the $P_1$ and $P_3$ approximations relative to the practically exact $P_{201}$. It can be seen that the $P_3$ is clearly superior to the simpler $P_1$ approximation. Especially in the vicinity of the light source near the upper boundary, the $P_1$ introduces large errors. However, the $P_3$ still underestimates the total fluence and therefore the total heat source strength per layer by $0.7-0.9\%$. Being able to quickly quantify these errors, in addition, enables us to correct them. This results in a weighting factor for the heat source strength in each layer, making sure that the total source strength in each layer, and consequently also in the whole medium, is correct. The corresponding fluence rates are also shown in Fig. 2.

**Figure 3:**
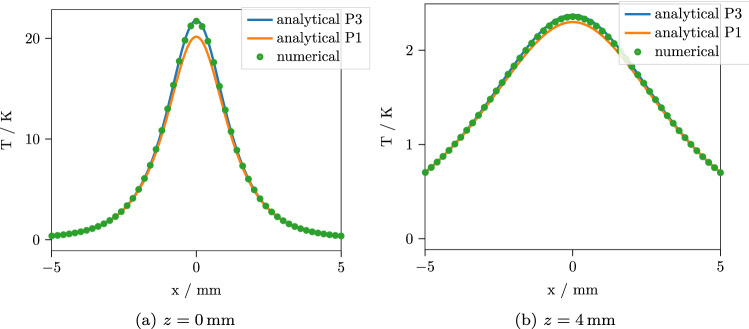
Temperature after $t=15\,\hbox {s}$ with normal incidence $\theta _1 = 0^\circ$ along the line $y=0$ for two different depths. The $P_3$ approximation shows a good agreement with the numerical reference solution, while the $P_1$ exhibits a considerable error.

**Figure 4:**
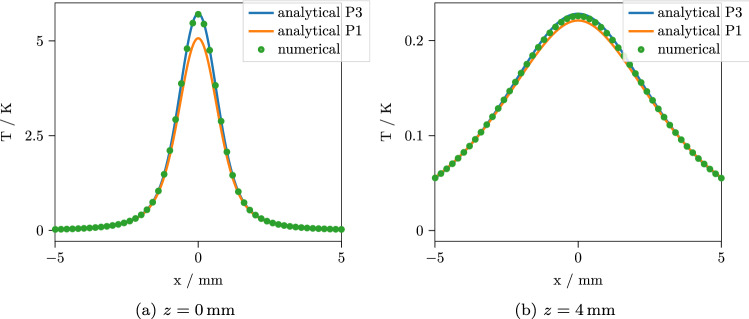
Temperature after $t=1\,\hbox {s}$ with normal incidence $\theta _1 = 0^\circ$ along the line $y=0$ for two different depths. The results are slightly less accurate for short times, but the $P_3$ approximation still agrees well with the numerical reference solution.

**Figure 5:**
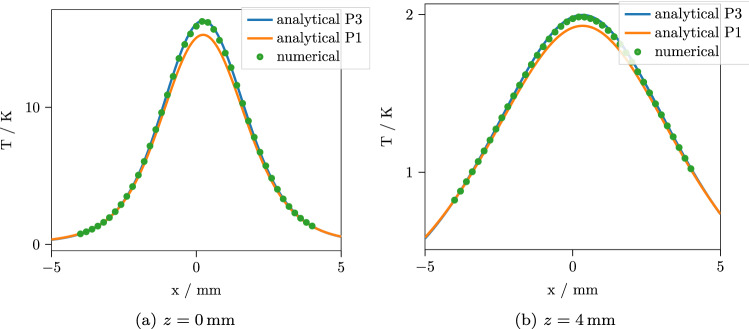
Temperature after $t={15}\,\hbox {s}$ with an incidence angle of $\theta _1 = 60^\circ$ along the line $y=0$ for two different depths. While the $P_3$ is still clearly superior to the $P_1$ approximation, neither of the approximations deteriorates much with oblique incidence.

**Figure 6:**
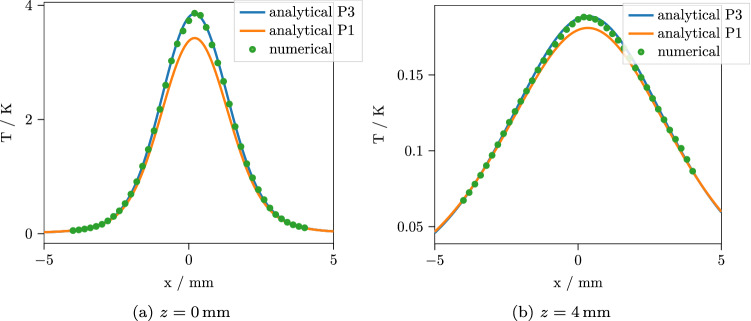
Temperature after $t={1}\,\hbox {s}$ with an incidence angle of $\theta _1 = 60^\circ$ along the line $y=0$ for two different depths. As for normal incidence, the accuracy is only slightly reduced for short times.

We implemented our solution in Python. The implementation includes the planar symmetric $P_N$ approximation solution, the 3D $P_1$ and $P_3$ approximation solutions, the solution of the heat equation with the $P_1$ and $P_3$ absorbance sources and the required numerical transform algorithms and is freely available together with the reference results of the numerical calculations^30^. The transform algorithms are basically the same that were used in a previous work^26^. For the comparison, we calculate the results along the line $y={0}\,\hbox {mm}$ at two depths $z=0\,\hbox {mm}$ and $z=4\,\hbox {mm}$, $t={1}\,\hbox {s}$ and $t={15}\,\hbox {s}$ after the source was switched on. Figures 3 and 4 show the results for normal incidence, while the results for an incident angle of $\theta _1 = 60^\circ$ are shown in Figs. 5 and 6. For each curve, 400 points were calculated. The performance depends, of course, on the efficiency of the numerical transforms and on the required accuracy. For the present calculations, we used 39 points for the inverse Laplace transform and $240 \times 80$ points for the inverse 2D Fourier transform, which resulted in a computation time of ${3.4}\,\hbox {s}$ for the $P_3$ approximation and ${2.7}\,\hbox {s}$ for the $P_1$ approximation per curve on a standard desktop PC. For the numerical solution, the Monte Carlo simulation took ${16}\,{\hbox {min}}$ and the subsequent finite element solution took ${44}\,{\hbox {min}}$ to complete with reasonable accuracy. As can be seen, the $P_3$ solution agrees quite well with the numerical result, while the $P_1$ predictably shows larger errors up to $7\%$. For short times, the errors are slightly larger for all cases. This is to be expected, since the errors in the $P_N$ absorbance are mostly contained in the high spatial frequency components. These are most prominent in the temperature solution for short times and then decrease due to the effects of heat conduction. To confirm that the remaining error of the $P_3$ approximation is indeed due to a slight misprediction of the heat source geometry instead of the interpolation or the finite element calculation, we repeated the numerical simulation with absorbance values computed from the $P_3$ approximation instead of the Monte Carlo simulation. For this comparison, we observed a practically exact agreement of the solutions.

## Conclusions

In conclusion, we derived a semianalytical solution for the heating of a scattering and absorbing three-layered medium by an incident beam of light, where the light transport is modeled using the $P_1$ and $P_3$ approximations to the RTE. We compared our solution to a purely numerical one and observed a good agreement in the case of the $P_3$ approximation for the light transport. The simpler $P_1$ approximation showed significantly larger errors and should not be used, given the modest advantage in calculation time compared to the $P_3$. For the comparison above, the semianalytical solution was several orders of magnitude faster than the numerical solution. An entirely fair comparison is of course difficult to perform, since the numerical simulation unavoidably also yields the absorbance for all positions inside the medium and the temperature for all positions and times. But in most situations, only a small subset of this data is actually required and this is where the semianalytical solution offers large improvements. Finally, some simple generalizations are possible for the heat conduction, since we only require the form of (36). For example, the equation for a moving heat source has the same form with a slightly different $\alpha$

51 $$\begin{aligned} \alpha ^{(n)}_u = \sqrt{\dfrac{\rho ^{(n)} c_p^{(n)}}{k^{(n)}} \left( s +iq(u_x \cos {\phi _q}+u_y\sin {\phi _q})\right) + q^2}, \end{aligned}$$

where $u_x$ and $u_y$ are the velocity components of the source in the x-y-plane.

## A Coefficients $A^{(n)}_2$ and $B^{(n)}_2$ for sources in the second layer

Here, we give closed form expressions for the coefficients $A^{(n)}_2$ and $B^{(n)}_2$ in Eq. (38). The coefficients $A^{(n)}_1$ and $B^{(n)}_1$ are contained in our previous work^26^ and $A^{(n)}_3$ and $B^{(n)}_3$ follow immediately by reversing the layer order. Adopting the same notation, the required coefficients follow from the system of equations

52 $$\begin{aligned}{}&\left( {\begin{matrix} (\gamma ^{(1)} - \frac{h_1}{k_z^{(1)}})e^{(1)} &{} -(\gamma ^{(1)} + \frac{h_1}{k_z^{(1)}}) &{} 0 &{} 0 &{} 0 &{} 0 \\ 1 &{} e^{(1)} &{} -e^{(2)} &{} -1 &{} 0 &{} 0 \\ k_z^{(1)}\gamma ^{(1)} &{} -k_z^{(1)}\gamma ^{(1)}e^{(1)} &{} -k_z^{(2)}\gamma ^{(2)}e^{(2)} &{} k_z^{(2)}\gamma ^{(2)} &{} 0 &{} 0\\ 0 &{} 0 &{} 1 &{} e^{(2)} &{} -e^{(3)} &{} -1\\ 0 &{} 0 &{} k_z^{(2)}\gamma ^{(2)} &{} -k_z^{(2)}\gamma ^{(2)}e^{(2)} &{} -k_z^{(3)}\gamma ^{(3)}e^{(3)} &{} k_z^{(3)}\gamma ^{(3)}\\ 0 &{} 0 &{} 0 &{} 0 &{} \gamma ^{(3)}+\frac{h_2}{k_z^{(3)}} &{} -(\gamma ^{(3)}-\frac{h_2}{k_z^{(3)}})e^{(3)} \end{matrix}}\right) \cdot \begin{pmatrix} A^{(1)}_2\\ B^{(1)}_2\\ A^{(2)}_2\\ B^{(2)}_2\\ A^{(3)}_2\\ B^{(3)}_2 \end{pmatrix} = \begin{pmatrix} 0\\ \frac{1}{k_z^{(2)}\gamma ^{(2)}}g_1\\ g_1\\ -\frac{1}{k_z^{(2)}\gamma ^{(2)}}g_2\\ g_2\\ 0 \end{pmatrix}, \end{aligned}$$

with

53 $$\begin{aligned} g_1= & {} f_2^{(1)}, \end{aligned}$$

54 $$\begin{aligned} g_2= & {} f_1^{(2)}. \end{aligned}$$

Again using the variables

55 $$\begin{aligned} p^{(n)}&= 1 + (e^{(n)})^2 , \end{aligned}$$

56 $$\begin{aligned} m^{(n)}&= 1 - (e^{(n)})^2 , \end{aligned}$$

the coefficients are found to be

57 $$\begin{aligned} A^{(1)}_2&= \frac{2}{M k_z^{(3)}} \left( \frac{h_1}{k_z^{(1)}}+\gamma ^{(1)}\right) \left[ \gamma ^{(3)}k_z^{(3)}(g_1-e^{(2)}g_2) (h_2 p^{(3)} + \gamma ^{(3)} k_z^{(3)} m^{(3)}) \right. \nonumber \\&\quad \left. + \gamma ^{(2)}k_z^{(2)}(g_1+e^{(2)}g_2) (h_2 m^{(3)} + \gamma ^{(3)} k_z^{(3)} p^{(3)}) \right] , \end{aligned}$$

58 $$\begin{aligned} B^{(1)}_2&= \frac{2 e^{(1)}}{M k_z^{(3)}} \left( \gamma ^{(1)}-\frac{h_1}{k_z^{(1)}}\right) \left[ \gamma ^{(3)}k_z^{(3)}(g_1-e^{(2)}g_2) (h_2 p^{(3)} + \gamma ^{(3)} k_z^{(3)} m^{(3)}) \right. \nonumber \\&\quad \left. + \gamma ^{(2)}k_z^{(2)}(g_1+e^{(2)}g_2) (h_2 m^{(3)} + \gamma ^{(3)} k_z^{(3)} p^{(3)}) \right] , \end{aligned}$$

59 $$\begin{aligned} A^{(2)}_2&= \frac{1}{M \gamma ^{(2)} k_z^{(1)}k_z^{(2)}k_z^{(3)}} \left[ -k_z^{(1)}\gamma ^{(1)}(g_1e^{(2)}-g_2)(h_1p^{(1)}+\gamma ^{(1)}k_z^{(1)}m^{(1)}) \right. \nonumber \\&\quad \left. + k_z^{(2)}\gamma ^{(2)}(g_1e^{(2)}+g_2)(h_1m^{(1)}+\gamma ^{(1)}k_z^{(1)}p^{(1)}) \right] \nonumber \\&\quad \times \left[ \gamma ^{(2)} k_z^{(2)}(h_2m^{(3)}+\gamma ^{(3)}k_z^{(3)}p^{(3)})-\gamma ^{(3)}k_z^{(3)}(h_2p^{(3)}+\gamma ^{(3)}k_z^{(3)}m^{(3)})\right] , \end{aligned}$$

60 $$\begin{aligned} B^{(2)}_2&= \frac{1}{M \gamma ^{(2)} k_z^{(1)}k_z^{(2)}k_z^{(3)}} \left[ -(\gamma ^{(1)})^2(k_z^{(1)})^2 m^{(1)} + \gamma ^{(2)}k_z^{(2)}h_1m^{(1)} \right. \nonumber \\&\quad \left. - \gamma ^{(1)}k_z^{(1)}p^{(1)}(h_1-\gamma ^{(2)}k_z^{(2)}) \right] \times \left[ k_z^{(3)}\gamma ^{(3)}(g_1-e^{(2)}g_2)(h_2p^{(3)}+\gamma ^{(3)}k_z^{(3)}m^{(3)}) \right. \nonumber \\&\quad \left. + k_z^{(2)}\gamma ^{(2)}(g_1+e^{(2)}g_2)(h_2m^{(3)}+\gamma ^{(3)}k_z^{(3)}p^{(3)}) \right] , \end{aligned}$$

61 $$\begin{aligned} A^{(3)}_2&= \frac{2e^{(3)}}{k_z^{(1)}} \left( \gamma ^{(3)}-\frac{h_2}{k_z^{(3)}} \right) \times \left[ -\gamma ^{(1)}k_z^{(1)}(e^{(2)}g_1-g_2)(h_1p^{(1)}+\gamma ^{(1)}k_z^{(1)}m^{(1)}) \right. \nonumber \\&\quad \left. + \gamma ^{(2)}k_z^{(2)}(e^{(2)}g_1+g_2)(h_1m^{(1)}+\gamma ^{(1)}k_z^{(1)}p^{(1)}) \right] , \end{aligned}$$

62 $$\begin{aligned} B^{(3)}_2&= \frac{2}{k_z^{(1)}} \left( \gamma ^{(3)}+\frac{h_2}{k_z^{(3)}} \right) \times \left[ -\gamma ^{(1)}k_z^{(1)}(e^{(2)}g_1-g_2)(h_1p^{(1)}+\gamma ^{(1)}k_z^{(1)}m^{(1)}) \right. \nonumber \\&\quad \left. + \gamma ^{(2)}k_z^{(2)}(e^{(2)}g_1+g_2)(h_1m^{(1)}+\gamma ^{(1)}k_z^{(1)}p^{(1)}) \right] . \end{aligned}$$

## B $P_1$ solution

For the $P_1$ approximation, we get only a single eigenvalue

63 $$\begin{aligned} \xi _1 = \dfrac{1}{\sqrt{3\sigma _0\sigma _1}} . \end{aligned}$$

The corresponding eigenvector has three components and can be found via the method of rotated reference frames

64 $$\begin{aligned} \Lambda ^1_{00}&= -\sqrt{3} \sigma _1, \end{aligned}$$

65 $$\begin{aligned} \Lambda ^1_{10}&= -\sqrt{q^2 + \frac{1}{{\xi _1}^2}}, \end{aligned}$$

66 $$\begin{aligned} \Lambda ^1_{11}&= -\frac{i}{\sqrt{2}} q. \end{aligned}$$

For the particular solution, the system (17) for the $P_1$ approximation reads

67 $$\begin{aligned} \begin{pmatrix} \sigma _0 &{} -\frac{\mu _c}{\sqrt{3}} &{} -\sqrt{\frac{2}{3}}iq \\ -\frac{\mu _c}{\sqrt{3}} &{} \sigma _1 &{} 0 \\ -\sqrt{\frac{1}{6}}iq &{} 0 &{} \sigma _1 \\ \end{pmatrix} \cdot \begin{pmatrix} \chi _{00} \\ \chi _{10} \\ \chi _{11} \end{pmatrix} = \begin{pmatrix} \frac{\mu _s}{\mu _0} (f_0-f_2) \frac{1}{\sqrt{4\pi }} \\ \mu _s (f_1-f_2) \frac{3}{\sqrt{4\pi }} \\ \mu _s (f_2-f_1) \frac{3}{\sqrt{8\pi }} \frac{\sqrt{1-\mu _0^2}}{\mu _0} \cos {\phi _q} \end{pmatrix}. \end{aligned}$$

This system can be solved analytically. Alternatively, the matrix can be LU-decomposed for an efficient numerical solution as

68 $$\begin{aligned} \begin{pmatrix} 1 &{} 0 &{}0 \\ 0 &{} 1 &{} 0 \\ -\frac{\sqrt{\frac{2}{3}}iq}{\sigma _1} &{} -\frac{\mu _c}{\sqrt{3}\sigma _1} &{} 1 \end{pmatrix} \cdot \begin{pmatrix} \sigma _1 &{} 0 &{} -\frac{iq}{\sqrt{6}} \\ 0 &{} \sigma _1 &{} -\frac{\mu _c}{\sqrt{3}} \\ 0 &{} 0 &{} \sigma _0+\frac{q^2-\mu _c^2}{3\sigma _1} \end{pmatrix} \cdot \begin{pmatrix} \chi _{11} \\ \chi _{10} \\ \chi _{00} \end{pmatrix} = \begin{pmatrix} \mu _s (f_2-f_1) \frac{3}{\sqrt{8\pi }} \frac{\sqrt{1-\mu _0^2}}{\mu _0} \cos {\phi _q} \\ \mu _s (f_1-f_2) \frac{3}{\sqrt{4\pi }} \\ \frac{\mu _s}{\mu _0} (f_0-f_2) \frac{1}{\sqrt{4\pi }} \end{pmatrix}. \end{aligned}$$
